# Design and Synthesis of Novel Isoxazole Tethered Quinone-Amino Acid Hybrids

**DOI:** 10.1155/2014/721291

**Published:** 2014-11-19

**Authors:** P. Ravi Kumar, Manoranjan Behera, M. Sambaiah, Venu Kandula, Nagaraju Payili, A. Jaya Shree, Satyanarayana Yennam

**Affiliations:** ^1^Department of Medicinal Chemistry, GVK Biosciences Pvt. Ltd., Plot No. 28, IDA, Nacharam, Hyderabad, Andhra Pradesh 500 076, India; ^2^Centre for Chemical Sciences & Technology, Institute of Science and Technology, Jawaharlal Nehru Technological University, Kukatpally, Hyderabad, Andhra Pradesh 500 072, India

## Abstract

A new series of isoxazole tethered quinone-amino acid hybrids has been designed and synthesized involving 1,3-dipolar cycloaddition reaction followed by an oxidation reaction using cerium ammonium nitrate (CAN). Using this method, for the first time various isoxazole tethered quinone-phenyl alanine and quinone-alanine hybrids were synthesized from simple commercially available 4-bromobenzyl bromide, propargyl bromide, and 2,5-dimethoxybenzaldehyde in good yield.

## 1. Introduction

Compounds containing the quinone group present an important class of biologically active molecules that are widespread in nature [1–3]. The discoveries of antibiotic [4, 5] and antitumor [6] properties assigned to several natural quinones have raised interest among scientists for use as pharmaceuticals. While antibiotics display an enormous diversity in chemical structures, quinone antibiotics such as Adriamycin, Mitomycin C, and Streptonigrin deserve special attention [7–10]. In this context, search of new molecules containing quinone moiety has always fascinated the organic as well as medicinal chemist.

Isoxazole derivatives are an important class of heterocyclic pharmaceuticals and bioactive natural products because of their significant and wide spectrum of biological activities, including potent and selective antagonism of the NMDA receptor and anti-HIV activity^.^ [11, 12]. It shows antihyperglycemic [13], analgesic [14], anti-inflammatory [15], antifungal [16], and antibacterial activity [17]. 3,5-Disubstituted isoxazole derivatives which are biological active include muscimol, dihydromuscimol, micafungin, and cycloserine [18, 19]. Unnatural amino acids, the nonproteinogenic *α*-amino acids that occur either naturally or chemically synthesized, have been used widely as chiral building block. They have been also used as molecular scaffolds in constructing combinatorial libraries [20]. They represent a powerful tool in drug discovery when incorporated into therapeutic peptidomimetics and peptide analogs [21]. The seminal work on the synthesis of unnatural amino acids has been done by O'Donnell and Maruoka independently, which accelerated the application of this class of amino acid for practical applications [22, 23].

Synthesis of hybrid natural products has gained momentum in recent years [24–26]. It is expected that combining features of more than one biologically active natural segment in a single molecule may result in pronounced pharmacological activity while retaining high diversity and biological relevance. There are a few reports describing the preparation of quinone-hybrid with other natural products. For example, quinone-amino acids [27], sugar-oxasteroid-quinone [28], quinone-annonaceous acetogenins [29], and conduritol-carba-sugar [30] hybrids have been described using different synthetic protocol.

**Figure 1:**
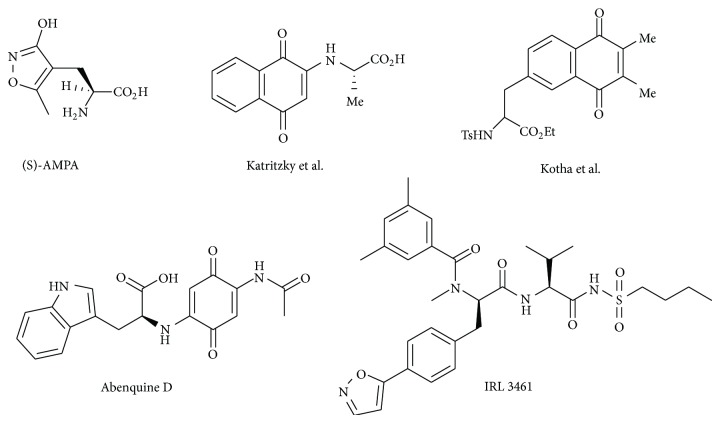
Selected examples of amino acid hybrids.

In our continuation endeavour to prepare novel hybrid molecules containing variety of natural products [31], we developed interest in the synthesis of novel isoxazole tethered quinone-amino acid hybrid natural products, and herein we report our initial results. Depending on the hybrid pattern, hybrid molecules containing amino acids and either quinone or isoxazole were prepared by various groups using different methods (Figure 1). For example, AMPA (*α*-amino-3-hydroxy-5-methyl-4-isoxazolepropionic acid) is a type of glutamatergic ion channels in the central nervous system which can be considered as isoxazole-amino acid hybrid. A series of novel AMPA analogues were prepared in order to evaluate it as drug candidates for neurological disorder [32]. Abenquine D is an amino acid quinone hybrid which is composed of an amino acid linked to an N-acetyl-amino benzoquinone. Abenquines A–D are new bioactive secondary metabolites found in the fermentation broth of* Streptomyces* sp. stain DB634 which was isolated from the soils of Chilean highland of Atacama desert. The abenquines show inhibitory activity against bacteria, dermatophytic fungi, and phosphodiesterase type** 4b** [33]. It is noteworthy to mention here that amino acid attached to the quinone is relevant to the enzyme inhibitory activity. Similarly, IRL 3461 is a potent and bifunctional ET_A_/ET_B_ endothelin antagonist. IRL 346a is an isoxazole-amino acid hybrid prepared from 4-methyl-acetophenone in nine steps synthetic protocol [34]. Katritzky et al. have prepared naphthoquinone-amino acid conjugates starting from naphthoquinone and L-amino acids by a Michael type mechanism in aqueous ethanol solution at RT in the presence of triethylamine [35]. Kotha group has used a “building block approach” to synthesize the quinone-amino acid hybrids through ethylene cross-enyne metathesis and Diels-Alder reaction as the key step [27]. But there are no reports of isoxazole tethered quinone amino acid hybrids as per the literature search. To the best of our knowledge, this is the first report on the synthesis of new series of isoxazole tethered quinone-amino acid hybrid natural products.

**Figure 2:**
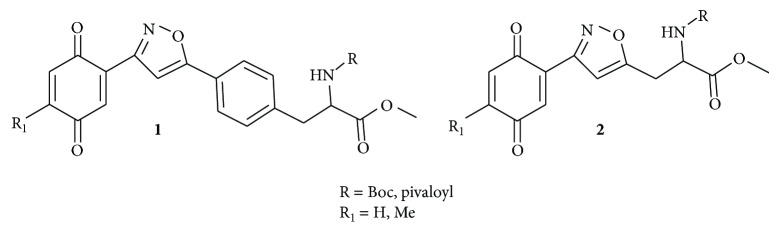
Isoxazole tethered quinone amino acid hybrid.

In view of the importance of these three classes of natural products, we have designed a new class of hybrid structures** 1** or** 2** (Figure 2) in an effort to combine the activity of amino acid moiety and the quinone unit using isoxazole ring as linker. These hybrids may have significant biological activity and so an efficient strategy to these hybrid molecules would allow us to construct diverse hybrid analogues.

## 2. Materials and Methods

All reactions were carried out in oven-dried glassware with magnetic stirrers under an argon atmosphere. THF was dried over Na/benzophenone and DCM was dried over CaH_2_. Commercially available chemicals were purchased from Sigma-Aldrich and Alfa Aesar. EtOAc and pet ether were distilled before use. All melting points were taken in open capillaries and are uncorrected. Analytical thin-layer chromatography (TLC) was performed on commercially available Merck TLC Silica gel 60 F_254_. Silica gel column chromatography was performed on silica gel 60 (spherical 100–200 *μ*m). FTIR spectra were recorded on Perkin-Elmer FT/IR-4000 spectrophotometer and only the characteristic peaks are reported. Mass spectra were scanned on a Shimadzu LCMS 2010 spectrometer. ^1^H NMR spectra were recorded on Varian-400 (400 MHz) spectrometer. Chemical shifts of ^1^H NMR spectra were reported relative to tetramethylsilane. ^13^C NMR spectra were recorded on Varian-400 (100 MHz) spectrometer. Chemical shifts of ^13^C NMR spectra were reported to be relative to CDCl_3_  (77.0). Splitting patterns were reported as s, singlet; d, doublet; t, triplet; q, quartet; m, multiplet; dd, double doublet; and br, broad.

### 2.1. Experimental Procedure for the Preparation of Methyl 2-((tert-Butoxycarbonyl)amino)-3-(4-((trimethylsilyl)ethynyl)phenyl)propanoate (**4a**)

To a solution of compound** 3a **(1.0 g, 2.80 mmol) in triethylamine (10 mL), PdCl_2_(PPh_3_)_2_ (0.098 g, 0.14 mmol), CuI (0.013 g, 0.07 mmol), and trimethylsilylacetylene (0.411 g, 4.20 mmol) were added under argon atmosphere and heated at 80°C in a sealed tube for 12 h. The progress of the reaction was monitored by TLC analysis (20% ethyl acetate/pet ether). After completion of the reaction, the reaction mixture was filtered. The filtrate was evaporated to give the crude product which was charged on silica gel column. The column was eluted with 20% ethyl acetate/pet ether to give the compound** 4a** (0.800 g, 76% yield) as light yellow liquid.

IR (KBr, cm^−1^): 3375, 2961, 2158, 1716, 1505, 1250, 1168, 865, 843. ^1^H NMR (400 MHz, CDCl_3_): *δ* = 7.42–7.36 (m, 2H), 7.06 (d,* J *= 7.8 Hz, 2H), 4.94 (d,* J *= 8.1 Hz, 1H), 4.57 (d,* J *= 7.4 Hz, 1H), 3.69 (s, 3H), 3.09 (td,* J *= 14.2, 6.1 Hz, 2H), 1.42 (s, 9H), 0.2 (s, 9H). MS (EI):* m*/*z* 375 (M + 1, 100).

### 2.2. Experimental Procedure for the Preparation of Methyl 2-Pivalamido-3-(4-((trimethylsilyl)ethynyl)phenyl)propanoate (**4b**)

To a solution of compound** 3b **(3.5 g, 10.26 mmol) in triethylamine (20 mL), PdCl_2_(PPh_3_)_2_ (0.359 g, 0.51 mmol), CuI (0.048 g, 0.25 mmol), and trimethylsilylacetylene (1.20 g, 12.31 mmol) were added under argon atmosphere and heated at 90°C in a sealed tube for 12 h. The progress of the reaction was monitored by TLC analysis (30% ethyl acetate/pet ether). After completion of the reaction, the reaction mixture was filtered. The filtrate was evaporated to give the crude reaction mixture which was charged on silica gel column. The column was eluted with 20% ethyl acetate/pet ether to give the compound** 4b** (1.8 g, 48% yield) as off-white solid.

m.p. 143–145°C. IR (KBr, cm^−1^): 3328, 2958, 2158, 1751, 1638, 1205, 841. ^1^H NMR (300 MHz, DMSO): *δ* = 7.40 (dd,* J *= 1.7, 7.8 Hz, 2H), 7.20 (dd,* J *= 10.3, 8.1 Hz, 2H), 4.53–4.38 (m, 1H), 3.61 (d,* J *= 1.7 Hz, 3H), 3.18–2.89 (m, 2H), 1.00 (d,* J *= 1.6 Hz, 9H), 0.2 (s, 9H). MS (EI):* m*/*z* 360 (M + 1, 100).

### 2.3. Experimental Procedure for the Preparation of Methyl 2-((tert-Butoxycarbonyl)amino)-3-(4-ethynylphenyl)propanoate (**5a**)

To a solution of compound** 4a **(0.800 g, 2.13 mmol) in THF (10 mL), 1 M TBAF in THF (4.26 mL, 4.26 mmol) was added at −70°C and stirred for 2 h. The progress of the reaction was monitored by TLC analysis (20% ethyl acetate/pet ether). After the reaction was complete, the reaction mixture was quenched with water (10 mL) and extracted with ethyl acetate thrice. The organic layers were combined and washed with water, brined, and dried over anhydrous Na_2_SO_4_. Evaporation of the solvent in high vacuum gave the compound** 5a** (0.650 g, 95% yield) as light yellow solid.

m.p. 94–97°C. IR (KBr, cm^−1^): 3355, 2974, 2103, 1739, 1682, 1519, 1170, 826. ^1^H NMR (400 MHz, CDCl_3_): *δ* = 7.42 (d,* J *= 7.8 Hz, 2H), 7.09 (d,* J *= 7.8 Hz, 2H), 4.96 (s, 1H), 4.58 (d,* J *= 7.8 Hz, 1H), 3.71 (d,* J *= 1.0 Hz, 3H), 3.21–2.94 (m, 3H), 1.42 (s, 9H). MS (EI):* m*/*z* 303 (M + 1, 100).

### 2.4. Experimental Procedure for the Preparation of Methyl 3-(4-Ethynylphenyl)-2-pivalamidopropanoate (**5b**)

To a solution of compound** 4b **(1.0 g, 3.84 mmol) in THF (20 mL), 1 M TBAF in THF (3.8 mL, 7.66 mmol) was added at −70°C and stirred for 2 h. The progress of the reaction was monitored by TLC analysis (20% ethyl acetate/pet ether). After the reaction was complete, the reaction mixture was quenched with water (20 mL) and extracted with ethyl acetate thrice. The organic layers were combined, washed with water, brined, and dried over anhydrous Na_2_SO_4_. Evaporation of the solvent in high vacuum gave the compound** 5b** (0.650 g, 82% yield) as off-white solid.

m.p. 65–68°C. IR (KBr, cm^−1^): 3326, 2957, 1750, 1737, 1637, 1522, 1202, 1116. ^1^H NMR (400 MHz, DMSO): *δ* = 7.39–7.33 (m, 2H), 7.27–7.20 (m, 2H), 4.46 (m, 1H), 4.12 (s, 1H), 3.61 (s, 3H), 3.18–2.90 (m, 2H), 1.00 (d,* J *= 2.2 Hz, 9H). MS (EI):* m*/*z* 288 (M + 1, 100).

### 2.5. Experimental Procedure for the Preparation of 2,5-Dimethoxybenzaldehyde Oxime (**7a**)

To a solution of compound** 6a **(1 g, 6.02 mmol) in MeOH (10 mL), NaOAc (0.98 g, 12.04 mmol) and NH_2_OH*·*HCl (0.62 g, 9.03 mmol) were added under nitrogen atmosphere. Then the reaction mixture was stirred at RT for 2 h. The progress of the reaction was monitored by TLC analysis (20% ethyl acetate/pet ether). After completion of the reaction, the solvent was evaporated, quenched with water (20 mL), and extracted with ethyl acetate thrice. The organic layers were combined, washed with water, brined, and dried over anhydrous Na_2_SO_4_. Evaporation of the solvent in high vacuum gave the compound** 7a** (1.0 g, 91% yield) as off-white solid.

m.p. 105–107°C. IR (KBr, cm^−1^): 3245, 2838, 1577, 1503, 1277, 1232, 1038, 970. ^1^H NMR (400 MHz, CDCl_3_): *δ* = 8.5 (s, 1H), 7.6–8.0 (br s, 1H), 7.3 (m, 1H), 6.9 (m, 1H), 6.8 (m, 1H), 3.8 (s, 3H), 3.7 (s, 3H). MS (EI):* m*/*z* 181 (M^+^, 100).

### 2.6. Experimental Procedure for the Preparation of 2,5-Dimethoxy-4-methylbenzaldehyde Oxime (**7b**)

To a solution of compound** 6b **(3.0 g, 16.6 mmol) in MeOH (30 mL), NaOAc (2.73 g, 33.3 mmol) and NH_2_OH*·*HCl (1.73 g, 24.9 mmol) were added under nitrogen atmosphere. Then the reaction mixture was stirred at RT for 2 h. The progress of the reaction was monitored by TLC analysis (20% ethyl acetate/pet ether). After completion of the reaction, the solvent was evaporated, quenched with water (20 mL), and extracted with ethyl acetate thrice. The organic layers were combined, washed with water, brined, and dried over anhydrous Na_2_SO_4_. Evaporation of the solvent in high vacuum gave the compound** 7b** (3.05 g, 93% yield) as off-white solid.

m.p. 125–129°C. IR (KBr, cm^−1^): 3187, 2995, 1613, 1405, 1212, 1045. ^1^H NMR (400 MHz, CDCl_3_): *δ* = 11.10 (s, 1H), 8.20 (s, 1H), 7.15 (s, 1H), 6.9 (s, 1H), 3.75 (s, 6H), 2.2 (s, 3H). MS (EI):* m*/*z* 195 (M^+^, 100).

### 2.7. Experimental Procedure for the Preparation of Methyl 2-((tert-Butoxycarbonyl)amino)-3-(4-(3-(2,5-dimethoxyphenyl)isoxazol-5-yl)phenyl)propanoate (**8a**)

To a solution of compound** 7a **(0.150 g, 0.83 mmol) in dichloromethane (8 mL), compound** 5a** (0.27 g, 0.91 mmol), triethylamine (0.12 g, 1.24 mmol), and NaOCl (9–12% in H_2_O, 8 mL) were added at 0°C under nitrogen atmosphere. Then the reaction mixture was stirred at RT for 12 h. The progress of the reaction was monitored by TLC analysis (30% ethyl acetate/pet ether). After completion of the reaction, the solvent was evaporated, quenched with water (20 mL), and extracted with dichloromethane. The organic layers were combined, washed with water, brined, and dried over anhydrous Na_2_SO_4_. Evaporation of the solvent in high vacuum gave the compound** 8a** (0.28 g, 71% yield) as light yellow liquid.

IR (KBr, cm^−1^): 3304, 2970, 2939, 1712, 1627, 1500, 1276, 1222, 1170, 1045. ^1^H NMR (300 MHz, CDCl_3_): *δ* = 7.78 (d,* J *= 8.0 Hz, 2H), 7.59–7.48 (m, 1H), 7.24 (s, 2H), 7.04 (s, 1H), 6.98 (t,* J *= 1.8 Hz, 2H), 5.00 (s, 1H), 4.62 (s, 1H), 3.97–3.63 (m, 9H), 3.22 (d,* J* = 3.1 Hz, 2H), 1.42 (s, 9H). MS (EI):* m*/*z* 482 (M + 1, 100).

### 2.8. Experimental Procedure for the Preparation of Methyl 3-(4-(3-(2,5-Dimethoxyphenyl)isoxazol-5-yl)phenyl)-2-pivalamidopropanoate (**8b**)

To a solution of compound** 7a **(0.2 g, 1.11 mmol) in dichloromethane (10 mL), compound** 5b** (0.35 g, 1.22 mmol), triethylamine (0.16 g, 1.66 mmol), and NaOCl (9–12% in water, 10 mL) were added at 0°C under nitrogen atmosphere. Then the reaction mixture was stirred at RT for 12 h. The progress of the reaction was monitored by TLC analysis (30% ethyl acetate/pet ether). After completion of the reaction, the solvent was evaporated, quenched with water (20 mL), and extracted with dichloromethane. The organic layers were combined, washed with water, brined, and dried over anhydrous Na_2_SO_4_. Evaporation of the solvent gives the crude reaction mixture which was charged on silica gel column. The column was eluted with 25% ethyl acetate/pet ether to give the compound** 8b** (0.3 g, 58% yield) as light yellow liquid.

IR (KBr, cm^−1^): 3437, 2926, 1623, 1275, 1260, 764, 750. ^1^H NMR (300 MHz, CDCl_3_): *δ* = 7.82–7.73 (m, 2H), 7.55–7.47 (m, 1H), 7.24–7.16 (m, 2H), 7.05 (s, 1H), 6.98 (t,* J *= 1.9 Hz, 2H), 6.12 (d,* J *= 7.5 Hz, 1H), 4.90 (dt,* J *= 7.5, 5.8 Hz, 1H), 3.89 (s, 3H), 3.84 (s, 3H), 3.81–3.74 (m, 3H), 3.33–3.07 (m, 2H), 1.17 (s, 9H). ^13^C NMR (100 MHz, CDCl_3_): *δ*_C_ 177.9, 172.0, 169.0, 160.4, 153.6, 151.6, 138.0, 129.8, 126.6, 125.8, 118.4, 117.3, 114.9, 113.5, 113.0, 112.4, 100.9, 56.2, 55.8, 52.7, 52.4, 42.7, 38.7, 38.6, 37.7, 29.6. HRMS (ESI): Calcd for C_26_H_31_N_2_O_6_ [M + H]^+^: 467.2182; Found: 467.2455.

### 2.9. Experimental Procedure for the Preparation of Methyl 2-((tert-Butoxycarbonyl)amino)-3-(4-(3-(2,5-dimethoxy-4-methylphenyl)isoxazol-5-yl)phenyl)propanoate (**8c**)

To a solution of compound** 7b **(0.2 g, 1.02 mmol) in dichloromethane (10 mL), compound** 5a** (0.34 g, 1.12 mmol), triethylamine (0.15 g, 1.53 mmol), and NaOCl (9–12% in H_2_O, 10 mL) were added at 0°C under nitrogen atmosphere. Then the reaction mixture was stirred at RT for 12 h. The progress of the reaction was monitored by TLC analysis (30% ethyl acetate/pet ether). After completion of the reaction, water (20 mL) was added and extracted with dichloromethane thrice. The organic layers were combined, washed with water, brined, and dried over anhydrous Na_2_SO_4_. The solvent was evaporated to give the crude reaction mixture which was charged on silica gel column. Elution of the column with 20% ethyl acetate/pet ether gave the compound** 8c** (0.41 g, 80% yield) as off-white solid.

m.p 147–150°C. IR (KBr, cm^−1^): 3373, 3149, 2932, 1742, 1702, 1520, 1216, 1044. ^1^H NMR (300 MHz, CDCl_3_): *δ* = 7.78 (d,* J *= 7.8 Hz, 2H), 7.45 (s, 1H), 7.26 (d,* J* = 1.4 Hz, 2H), 7.06 (s, 1H), 6.85 (s, 1H), 5.03 (d,* J* = 8.4 Hz, 1H), 4.63 (q,* J* = 6.7 Hz, 1H), 3.87 (d,* J* = 4.6 Hz, 6H), 3.73 (s, 3H), 3.17 (td,* J* = 16.2, 15.0, 5.8 Hz, 2H), 2.28 (s, 3H), 1.42 (s, 9H). ^13^C NMR (100 MHz, CDCl_3_): *δ*_C_ 172.0, 168.9, 160.5, 155.0, 151.9, 151.1, 138.1, 130.0, 129.8, 126.6, 125.9, 115.3, 115.0, 110.3, 100.9, 80.0, 56.3, 55.9, 54.2, 52.3, 38.3, 28.2, 16.6. MS (EI)* m*/*z* 496 (M + 1, 100). HRMS (ESI): Calcd for C_27_H_33_N_2_O_7_ [M + H]^+^: 497.1882; Found: 497.2288.

### 2.10. Experimental Procedure for the Preparation of Methyl 3-(4-(3-(2,5-Dimethoxy-4-methylphenyl)isoxazol-5-yl)phenyl)-2-pivalamidopropanoate (**8d**)

To a solution of compound** 7b **(0.15 g, 0.76 mmol) in dichloromethane (10 mL), compound** 5b** (0.24 g, 0.84 mmol), triethylamine (0.116 g, 1.15 mmol), and NaOCl (9–12% in water, 10 mL) were added at 0°C under nitrogen atmosphere. Then the reaction mixture was stirred at RT for 12 h. The progress of the reaction was monitored by TLC analysis (30% ethyl acetate/pet ether). After completion of the reaction, water (10 mL) was added and the reaction mixture was extracted with dichloromethane thrice. The organic layers were combined, washed with water, brined, and dried over anhydrous Na_2_SO_4_. The solvent was evaporated to give the crude reaction which was charged on silica gel column. Elution of the column with 25% ethyl acetate/pet ether gave the compound** 8d** (0.31 g, 83% yield) as light yellow liquid.

IR (KBr, cm^−1^): 3444, 2929, 1637, 1473, 1275, 1261, 1215, 764. ^1^H NMR (300 MHz, DMSO): *δ* = 7.81 (dd,* J* = 13.0, 8.0 Hz, 3H), 7.41 (d,* J* = 8.1 Hz, 2H), 7.32 (d,* J* = 3.3 Hz, 2H), 7.07 (s, 1H), 4.51 (s, 1H), 3.85 (s, 3H), 3.80 (s, 3H), 3.64 (s, 3H), 3.24–2.96 (m, 2H), 2.20 (d,* J* = 19.4 Hz, 3H), 1.02 (s, 9H). MS (EI):* m*/*z* 480 (M + 1, 100).

### 2.11. Experimental Procedure for the Preparation of Methyl 2-((tert-Butoxycarbonyl)amino)-3-(4-(3-(3,6-dioxocyclohexa-1,4-dien-1-yl)isoxazol-5-yl)phenyl)propanoate (**9a**)

To a solution of compound** 8a **(0.15 g, 0.31 mmol) in acetonitrile (6 mL) and H_2_O (1 mL), CAN (0.511 g, 0.93 mmol) was added and the reaction mixture was stirred at RT for 1 h. The progress of the reaction was monitored by TLC analysis (30% ethyl acetate/pet ether). After completion of the reaction, water (10 mL) was added and extracted with ethyl acetate thrice. The organic layers were combined, washed with water, brined, and dried over anhydrous Na_2_SO_4_. Evaporation of the solvent in high vacuum gave the compound** 9a **(0.12 g, 85% yield) as yellow solid.

m.p. 125–127°C. IR (KBr, cm^−1^): 3355, 2979, 1743, 1720, 1654, 1522, 1288, 1251, 1167. ^1^H NMR (300 MHz, CDCl_3_): *δ* = 7.77 (d,* J* = 7.9 Hz, 2H), 7.48 (d,* J* = 1.9 Hz, 1H), 7.26 (d,* J* = 0.9 Hz, 2H), 7.12 (d,* J* = 0.9 Hz, 1H), 6.96–6.86 (m, 2H), 5.02 (s, 1H), 4.63 (s, 1H), 3.74 (d,* J* = 1.0 Hz, 3H), 3.29–3.02 (m, 2H), 1.42 (s, 9H). ^13^C NMR (100 MHz, CDCl_3_): *δ*_C_ 186.8, 185.2, 171.9, 170.7, 156.5, 154.9, 139.0, 136.8, 136.6, 134.4, 133.3, 130.0, 126.0, 125.7, 100.7, 80.1, 54.2, 52.3, 38.3, 28.2. HRMS (ESI): Calcd for C_24_H_24_N_2_O_7_ [M + H]^+^: 453.1662; Found: 453.1640.

### 2.12. Experimental Procedure for the Preparation of Methyl 3-(4-(3-(3,6-Dioxocyclohexa-1,4-dien-1-yl)isoxazol-5-yl)phenyl)-2-pivalamidopropanoate (**9b**)

To a solution of compound** 8b **(0.2 g, 0.42 mmol) in acetonitrile (8 mL) and H_2_O (2 mL), CAN (0.705 g, 1.28 mmol) was added and the reaction mixture was stirred at RT for 1 h. The progress of the reaction was monitored by TLC analysis (30% ethyl acetate/pet ether). After completion of the reaction, water (20 mL) was added and extracted with ethyl acetate thrice. The organic layers were combined, washed with water, brined, and dried over anhydrous Na_2_SO_4_. Evaporation of the solvent in high vacuum gave the compound** 9b **(0.136 g, 72% yield) as yellow solid.

m.p. 115–117°C. IR (KBr, cm^−1^): 3321, 2958, 1749, 1656, 1640, 1531, 1286, 1199, 1107. ^1^H NMR (300 MHz, CDCl_3_) *δ* = 7.76 (d,* J* = 7.9 Hz, 2H), 7.47 (d,* J* = 2.2 Hz, 1H), 7.23 (d,* J* = 7.9 Hz, 2H), 7.12 (s, 1H), 6.90 (d,* J* = 2.7 Hz, 2H), 6.13 (d,* J* = 7.7 Hz, 1H), 4.90 (q,* J* = 6.1 Hz, 1H), 3.77 (s, 3H), 3.37–3.04 (m, 2H), 1.17 (s, 9H). ^13^C NMR (100 MHz, CDCl_3_): *δ*_C_ 186.8, 185.1, 177.9, 172.0, 170.6, 156.5, 138.9, 136.8, 136.6, 134.4, 133.3, 130.0, 125.9, 125.7, 100.7, 52.7, 52.4, 38.6, 37.7, 27.3. HRMS (ESI): Calcd for C_24_H_25_N_2_O_6_ [M + H]^+^: 437.1713; Found: 437.1879.

### 2.13. Experimental Procedure for the Preparation of Methyl 2-((tert-Butoxycarbonyl)amino)-3-(4-(3-(4-methyl-3,6-dioxocyclohexa-1,4-dien-1-yl)isoxazol-5-yl)phenyl)propanoate (**9c**)

To a solution of compound** 8c **(0.23 g, 0.46 mmol) in acetonitrile (10 mL) and H_2_O (2 mL), CAN (0.76 g, 1.39 mmol) was added and the reaction mixture was stirred at RT for 1 h. The progress of the reaction was monitored by TLC analysis (30% ethyl acetate/pet ether). After completion of the reaction, water (20 mL) was added and extracted with ethyl acetate thrice. The organic layers were combined, washed with water, brined, and dried over anhydrous Na_2_SO_4_. Evaporation of the solvent in high vacuum gave the compound** 9c **(0.205 g, 95% yield) as yellow solid.

m.p. 153–155°C. IR (KBr, cm^−1^): 3364, 2979, 1732, 1693, 1660, 1524, 1252, 1170, 1020. ^1^H NMR (300 MHz, CDCl_3_): *δ* = 7.83–7.69 (m, 2H), 7.45 (s, 1H), 7.34–7.20 (m, 2H), 7.11 (s, 1H), 6.74 (q,* J* = 1.5 Hz, 1H), 5.04 (d,* J* = 8.1 Hz, 1H), 4.63 (q,* J* = 6.7 Hz, 1H), 3.74 (s, 3H), 3.29–3.00 (m, 2H), 2.13 (d,* J* = 1.6 Hz, 3H), 1.42 (s, 9H). ^13^C NMR (100 MHz, CDCl_3_): *δ*_C_ 187.4, 185.4, 171.9, 170.5, 156.5, 154.9, 146.2, 138.9, 134.3, 133.5, 133.4, 130.0, 126.0, 125.7, 100.8, 80.1, 54.2, 52.3, 38.3, 28.2, 15.5. HRMS (ESI): Calcd for C_25_H_27_N_2_O_7_ [M + H]^+^: 467.1818; Found: 467.1611.

### 2.14. Experimental Procedure for the Preparation of Methyl 3-(4-(3-(4-Methyl-3,6-dioxocyclohexa-1,4-dien-1-yl)isoxazol-5-yl)phenyl)-2-pivalamidopropanoate (**9d**)

To a solution of compound** 8d **(0.3 g, 0.62 mmol) in acetonitrile (12 mL) and H_2_O (3 mL), CAN (1.02 g, 1.86 mmol) was added and the reaction mixture was stirred at RT for 1 h. The progress of the reaction was monitored by TLC analysis (30% ethyl acetate/pet ether). After completion of the reaction, water (20 mL) was added and extracted with ethyl acetate thrice. The organic layers were combined, washed with water, brined, and dried over anhydrous Na_2_SO_4_. Evaporation of the solvent in high vacuum gave the compound** 9d **(0.25 g, 89% yield) as yellow solid.

m.p. 130–132°C. IR (KBr, cm^−1^): 3379, 2959, 2924, 1734, 1657, 1237, 1020, 807. ^1^H NMR (300 MHz, CDCl_3_): *δ* = 7.82–7.68 (m, 2H), 7.45 (d,* J *= 1.3 Hz, 1H), 7.22 (d,* J* = 7.9 Hz, 2H), 7.12 (d,* J* = 1.0 Hz, 1H), 6.74 (q,* J* = 1.4 Hz, 1H), 6.12 (d,* J* = 7.4 Hz, 1H), 4.90 (q,* J* = 6.2 Hz, 1H), 3.76 (d,* J* = 0.9 Hz, 3H), 3.34–3.05 (m, 2H), 2.18–2.07 (m, 3H), 1.17 (d,* J* = 0.9 Hz, 9H). ^13^C NMR (100 MHz, CDCl_3_): *δ*_C_ 187.3, 185.4, 177.9, 172.0, 170.4, 156.5, 146.2, 138.8, 134.2, 133.5, 133.4, 130.0, 125.9, 125.8, 100.9, 52.7, 52.4, 38.6, 37.7, 27.3, 15.5. HRMS (ESI): Calcd for C_25_H_27_N_2_O_7_ [M + H]^+^: 467.1818; Found: 467.1611.

### 2.15. Experimental Procedure for the Preparation of (3-(2,5-Dimethoxyphenyl)isoxazol-5-yl)methanol (**15a**)

To a solution of compound** 7a **(2 g, 11.11 mmol) in ethyl acetate (20 mL), compound** 14** (2.01 g, 16.66 mmol), N-chlorosuccinamide (2.21 g, 16.66 mmol), and NaHCO_3_ (1.86 g, 22.22 mmol) were added and the reaction mixture was refluxed for 16 h. The progress of the reaction was monitored by TLC analysis (30% ethyl acetate/pet ether). Then, water (20 mL) was added and the reaction mixture was extracted with ethyl acetate. The combined organic layer was washed with water, brined, and dried over anhydrous Na_2_SO_4_. Evaporation of the solvent gave the crude product which was purified by column chromatography to give the compound** 15a** (2.1 g, 80% yield) as white solid.

m.p. 69–73°C. IR (KBr, cm^−1^): 3330, 2943, 1709, 1510, 1295, 1225, 1036. ^1^H NMR (400 MHz, CDCl_3_): *δ* = 7.45 (d,* J* = 2.9 Hz, 1H), 7.01–6.90 (m, 2H), 6.79 (s, 1H), 4.82 (d,* J* = 6.3 Hz, 2H), 3.83 (d,* J* = 12.0 Hz, 6H), 2.19 (t,* J* = 6.5 Hz, 1H). MS (EI):* m*/*z* 235 (M + 1, 100).

### 2.16. Experimental Procedure for the Preparation of (3-(2,5-Dimethoxy-4-methylphenyl)isoxazol-5-yl)methanol (**15b**)

To a solution of compound** 7b **(1.0 g, 5.12 mmol) in ethyl acetate (20 mL), compound** 14** (0.93 g, 7.69 mmol), N-chlorosuccinamide (1.02 g, 7.69 mmol), and NaHCO_3_ (0.861 g, 10.2 mmol) were added and refluxed for 16 hr. The progress of the reaction was monitored by TLC analysis (30% Ethyl acetate/pet ether). Then, water (20 mL) was added and the reaction mixture was extracted with ethyl acetate. The combined organic layer was washed with water, brined, and dried over anhydrous Na_2_SO_4_. Evaporation of the solvent gave the crude product which was purified by column chromatography to give the compound** 15b** (1.0 g, 90% yield) as off-white solid.

m.p. 58–62°C. IR (KBr, cm^−1^): 3426, 2940, 2129, 1715, 1216, 1038. ^1^H NMR (300 MHz, DMSO): *δ* = 11.05 (s, 1H), 7.27 (s, 1H), 7.05 (s, 1H), 6.74 (s, 1H), 4.60 (d,* J* = 0.8 Hz, 2H), 3.80 (d,* J* = 10.2 Hz, 6H), 2.21 (s, 3H). MS (EI):* m*/*z* 250 (M + 1, 100).

### 2.17. Experimental Procedure for the Preparation of 5-(Bromomethyl)-3-(2,5-dimethoxyphenyl)isoxazole (**16a**)

To a solution of compound** 15a **(1.5 g, 6.38 mmol) in dichloromethane (15 mL), phosphorous tribromide (2.59 g, 9.57 mmol) was added at 0°C under nitrogen atmosphere. Then the reaction mixture was stirred at RT for 16 hr. The progress of the reaction was monitored by TLC analysis (30% ethyl acetate/pet ether). Then, water (10 mL) was added and the reaction mixture was extracted with dichloromethane. The combined organic layer was washed with water, brined, and dried over anhydrous Na_2_SO_4_. The solvent was evaporated and the crude reaction mixture was purified by column chromatography to give the compound** 16a** (1.2 g, 63% yield) as white solid.

m.p. 71–75°C. IR (KBr, cm^−1^): 3432, 2925, 1852, 1603, 1465, 1270, 1021. ^1^H NMR (400 MHz, CDCl_3_): *δ* = 7.47 (d,* J* = 3.0 Hz, 1H), 7.02–6.91 (m, 2H), 6.87 (s, 1H), 4.52 (s, 2H), 3.84 (d,* J* = 15.1 Hz, 6H). MS (EI):* m*/*z* 297 (M + 1, 100).

### 2.18. Experimental Procedure for the Preparation of 5-(Bromomethyl)-3-(2,5-dimethoxy-4-methylphenyl)isoxazole (**16b**)

To a solution of compound** 15b **(1.0 g, 4.0 mmol) in dichloromethane (20 mL), phosphorous tribromide (1.62 g, 6.0 mmol) was added at 0°C under nitrogen atmosphere. Then the reaction mixture was stirred at RT for 16 hr. The progress of the reaction was monitored by TLC analysis (30% ethyl acetate/pet ether). Then, water (20 mL) was added and the reaction mixture was extracted with dichloromethane. The combined organic layer was washed with water, brined, and dried over anhydrous Na_2_SO_4_. The solvent was evaporated and the crude reaction mixture was purified by column chromatography to give the compound** 16b** (1.0 g, 80% yield) as brown solid.

m.p. 65–68°C. IR (KBr, cm^−1^): 3445, 2936, 1716, 1471, 1285, 1218, 1042. ^1^H NMR (400 MHz, CDCl_3_): *δ* = 7.39 (s, 1H), 6.82 (d,* J* = 6.4 Hz, 2H), 4.82 (dd,* J* = 6.5 Hz, 2H), 3.85 (s, 6H), 2.27 (s, 3H). MS (EI):* m*/*z* 311 (M + 1, 100).

### 2.19. Experimental Procedure for the Preparation of Methyl 3-(3-(2,5-Dimethoxyphenyl)isoxazol-5-yl)-2-((diphenylmethylene)amino)propanoate (**17a**)

To a solution of compound** 10 **(1.12 g, 4.44 mmol) in acetonitrile (20 mL), K_2_CO_3_ (2.78 g, 20.2 mmol) was added under nitrogen atmosphere and stirred at RT for 1 hr. Then compound** 16a** (1.2 g, 4.04 mmol) was added and the reaction mixture was refluxed for 16 hr. The progress of the reaction was monitored by TLC analysis (20% ethyl acetate/pet ether). Then, reaction mixture was filtered and filtrate was evaporated. The crude reaction mixture was purified by column chromatography to give the compound** 17a** (1.5 g, 74% yield) as light yellow liquid.

IR (KBr, cm^−1^): 3292, 2952, 1740, 1510, 1276, 1227, 1043. ^1^H NMR (300 MHz, CDCl_3_): *δ* = 7.63 (d,* J* = 6.9 Hz, 2H), 7.44–7.28 (m, 7H), 7.04–6.83 (m, 4H), 6.52 (s, 1H), 4.47 (dd,* J* = 7.5, 5.8 Hz, 1H), 3.78 (d,* J* = 8.5 Hz, 6H), 3.59 (s, 3H), 3.53–3.41 (m, 2H). MS (EI):* m*/*z* 470 (M + 1, 100).

### 2.20. Experimental Procedure for the Preparation of Methyl 3-(3-(2,5-Dimethoxy-4-methylphenyl)isoxazol-5-yl)-2-((diphenylmethylene)amino)propanoate (**17b**)

To a solution of compound** 10 **(0.9 g, 3.55 mmol) in acetonitrile (20 mL), K_2_CO_3_ (2.45 g, 17.7 mmol) was added under nitrogen atmosphere and stirred at RT for 1 hr. Then compound** 16b** (1.21 g, 3.91 mmol) was added and the reaction mixture was refluxed for 16 hr. The progress of the reaction was monitored by TLC analysis (20% ethyl acetate/pet ether). Then, reaction mixture was filtered and filtrate was evaporated. The crude reaction mixture was purified by column chromatography to give the compound** 17b** (1.1 g, 69% yield) as off-white solid.

m.p. 142–146°C. IR (KBr, cm^−1^): 3447, 2949, 1736, 1284, 1213, 1041. ^1^H NMR (300 MHz, CDCl_3_): *δ* = 7.63 (d,* J* = 8.2 Hz, 2H), 7.49–7.24 (m, 7H), 7.04–6.92 (m, 2H), 6.75 (s, 1H), 6.53 (s, 1H), 4.61–4.36 (m, 1H), 3.79 (dd,* J* = 16.5 Hz, 6H), 3.59 (d,* J* = 0.9 Hz, 3H), 3.54–3.35 (m, 2H), 2.24 (s, 3H). MS (EI):* m*/*z* 484 (M + 1, 100).

### 2.21. Experimental Procedure for the Preparation of Methyl 2-Amino-3-(3-(2,5-dimethoxyphenyl)isoxazol-5-yl)propanoate (**18a**)

To a solution of compound** 17a **(1.5 g, 3.19 mmol) in diethyl ether (20 mL), 1 M HCl (20 mL) was added at 0°C. Then the reaction mixture was stirred at RT for 16 hr. The progress of the reaction was monitored by TLC analysis (10% methanol/chloroform). The layers were separated and the aqueous layer was basified with aqueous ammonia until PH 10 and extracted with ethyl acetate. The combined organic layers were washed with water, brined, and dried over anhydrous Na_2_SO_4_. Evaporation of the solvent gave the compound** 18a** (0.850 g, 87% yield) as pale yellow liquid.

IR (KBr, cm^−1^): 3383, 2953, 1738, 1602, 1471, 1227, 1042. ^1^H NMR (300 MHz, DMSO): *δ* = 7.27 (d,* J* = 3.0 Hz, 1H), 7.17–6.97 (m, 2H), 6.67 (s, 1H), 3.78 (d,* J* = 17.1 Hz, 7H), 3.64 (s, 3H), 3.07 (qd,* J* = 15.1, 6.6 Hz, 2H), 1.99 (s, 2H). MS (EI):* m*/*z* 306 (M + 1, 100).

### 2.22. Experimental Procedure for the Preparation of Methyl 2-Amino-3-(3-(2,5-dimethoxy-4-methylphenyl)isoxazol-5-yl)propanoate (**18b**)

To a solution of compound** 17b **(0.5 g, 1.03 mmol) in diethyl ether (10 mL), 1 M HCl (10 mL) was added at 0°C. Then the reaction mixture was stirred at RT for 16 hr. The progress of the reaction was monitored by TLC analysis (10% methanol/chloroform). The layers were separated and the aqueous layer was basified with aqueous ammonia until PH 10 and extracted with ethyl acetate. The combined organic layer was washed with water, brined, and dried over anhydrous Na_2_SO_4_. Evaporation of the solvent gave the compound** 18b** (0.27 g, 82% yield) as off-white solid.

m.p. 218–221°C. IR (KBr, cm^−1^): 3468, 2838, 1741, 1472, 1250, 1220, 1041. ^1^H NMR (300 MHz, DMSO): *δ* = 8.58 (s, 2H), 7.28 (s, 1H), 7.06 (s, 1H), 6.82 (s, 1H), 4.53 (t,* J* = 6.1 Hz, 1H), 3.93–3.65 (m, 9H), 3.41 (d,* J* = 6.2 Hz, 2H), 2.22 (s, 3H). MS (EI):* m*/*z* 320 (M + 1, 100).

### 2.23. Experimental Procedure for the Preparation of Methyl 2-((tert-Butoxycarbonyl)amino)-3-(3-(2,5-dimethoxyphenyl)isoxazol-5-yl)propanoate (**19a**)

To a solution of compound** 18a **(0.300 g, 0.98 mmol) in dichloromethane (15 mL), triethylamine (0.19 g, 1.98 mmol) was added. Then (Boc)_2_O (0.23 g, 1.07 mmol) was added and the reaction mixture was stirred at RT for 16 hr. The progress of the reaction was monitored by TLC analysis (30% ethyl acetate/pet ether). Then, water (10 mL) was added and the reaction mixture was extracted with dichloromethane. The combined organic layer was washed with water, brined, and dried over anhydrous Na_2_SO_4_. Evaporation of the solvent gave the crude compound which was purified by column chromatography to give the compound** 19a** (0.380 g, 95% yield) as light brown solid.

m.p. 110–113°C. IR (KBr, cm^−1^): 3372, 2948, 1742, 1690, 1524, 1274, 1220, 1025. ^1^H NMR (300 MHz, CDCl_3_): *δ* = 7.44 (d,* J* = 2.8 Hz, 1H), 7.01–6.88 (m, 2H), 6.62 (s, 1H), 5.24 (s, 1H), 4.70 (s, 1H), 3.90–3.72 (m, 9H), 3.37 (d,* J* = 6.0 Hz, 2H), 1.44 (s, 9H). ^13^C NMR (100 MHz, CDCl_3_): *δ*_C_ 171.2, 167.4, 159.9, 155.0, 153.6, 151.5, 118.2, 117.3, 113.4, 113.0, 104.5, 80.2, 56.1, 55.8, 52.6, 52.1, 29.7, 28.2. MS (EI):* m*/*z* 406 (M + 1, 100). HRMS (ESI) Calcd for C_20_H_26_N_2_O_7_ [M + H]^+^: 407.1818; Found: 407.1743.

### 2.24. Experimental Procedure for the Preparation of Methyl 3-(3-(2,5-Dimethoxyphenyl)isoxazol-5-yl)-2-pivalamidopropanoate (**19b**)

To a solution of compound** 18a **(0.300 g, 0.98 mmol) in DCM (15 mL), dimethylaminopyridine (0.012 g, 0.098 mmol) was added. Then pivaloyl chloride (0.356 g, 2.94 mmol) was added and the reaction mixture was stirred at RT for 16 hr. The progress of the reaction was monitored by TLC analysis (30% ethyl acetate/pet ether). Then, water (10 mL) was added and the reaction mixture was extracted with dichloromethane. The combined organic layer was washed with water, brined, and dried over anhydrous Na_2_SO_4_. Evaporation of the solvent gave the crude compound which was purified by column chromatography to give the compound** 19b** (0.300 g, 78% yield) as light yellow liquid.

IR (KBr, cm^−1^): 3366, 2959, 1745, 1651, 1511, 1267, 1227, 1043. ^1^H NMR (300 MHz, CDCl_3_): *δ* = 7.44 (d,* J* = 2.8 Hz, 1H), 7.03–6.85 (m, 2H), 6.59 (d,* J* = 2.3 Hz, 1H), 6.38 (d,* J* = 7.0 Hz, 1H), 5.00–4.80 (m, 1H), 3.95–3.70 (m, 9H), 3.56–3.28 (m, 2H), 1.22 (d,* J* = 2.3 Hz, 9H). ^13^C NMR (100 MHz, CDCl_3_): *δ*_C_ 178.2, 171.1, 167.3, 159.9, 153.6, 151.5, 118.1, 117.4, 113.3, 112.9, 104.7, 56.0, 55.8, 52.8, 50.8, 38.6, 28.9, 27.3, 27.0. MS (EI):* m*/*z* 390 (M + 1, 100).

### 2.25. Experimental Procedure for the Preparation of Methyl 2-((tert-Butoxycarbonyl)amino)-3-(3-(2,5-dimethoxy-4-methylphenyl)isoxazol-5-yl)propanoate (**19c**)

To a solution of compound** 18b **(0.2 g, 0.62 mmol) in dichloromethane (10 mL), triethyl amine (0.12 g, 1.25 mmol) was added. Then (Boc)_2_O (0.15 g, 0.68 mmol) was added and the reaction mixture was stirred at RT for 16 hr. The progress of the reaction was monitored by TLC analysis (30% ethyl acetate/pet ether). Then, water (10 mL) was added and the reaction mixture was extracted with dichloromethane. The combined organic layer was washed with water, brined, and dried over anhydrous Na_2_SO_4_. Evaporation of the solvent gave the crude compound which was purified by column chromatography to give the compound** 19c** (0.25 g, 95% yield) as off-white solid.

m.p. 103–107°C. IR (KBr, cm^−1^): 3344, 2928, 2846, 1733, 1677, 1526, 1219, 1048. ^1^H NMR (300 MHz, DMSO): *δ* = 7.47 (d,* J* = 8.3 Hz, 1H), 7.26 (s, 1H), 7.04 (s, 1H), 6.68 (d,* J* = 4.3 Hz, 1H), 4.39 (t,* J* = 9.2 Hz, 1H), 3.91–3.73 (m, 6H), 3.65 (d,* J* = 10.3 Hz, 3H), 3.28–3.04 (m, 2H), 2.21 (s, 3H), 1.35 (s, 9H). ^13^C NMR (100 MHz, DMSO-d_6_): *δ*_C_ 171.9, 168.7, 159.2, 155.2, 151.2, 150.7, 129.3, 115.3, 114.6, 109.6, 109.5, 103.6, 78.5, 56.1, 55.5, 52.1, 51.9, 28.0, 26.8, 16.2. MS (EI):* m*/*z* 420 (M + 1, 100). HRMS (ESI) Calcd for C_21_H_29_N_2_O_7_ [M + H]^+^: 421.1975; Found: 421.1988.

### 2.26. Experimental Procedure for the Preparation of Methyl 3-(3-(2,5-Dimethoxy-4-methylphenyl)isoxazol-5-yl)-2-pivalamidopropanoate (**19d**)

To a solution of compound** 18b **(0.3 g, 0.93 mmol) in dichloromethane (10 mL), dimethylaminopyridine (0.011 g, 0.093 mmol) was added. Then pivaloyl chloride (0.34 g, 2.81 mmol) was added and the reaction mixture was stirred at RT for 16 hr. The progress of the reaction was monitored by TLC analysis (30% ethyl acetate/pet ether). Then, water (10 mL) was added and the reaction mixture was extracted with dichloromethane. The combined organic layer was washed with water, brined, and dried over anhydrous Na_2_SO_4_. Evaporation of the solvent gave the crude compound which was purified by column chromatography to give the compound** 19d** (0.31 g, 82% yield) as off-white solid.

m.p. 116–120°C. IR (KBr, cm^−1^): 3323, 2963, 1741, 1531, 1431, 1217, 1041. ^1^H NMR (300 MHz, DMSO): *δ* = 7.98 (d,* J* = 8.0 Hz, 1H), 7.25 (s, 1H), 7.03 (s, 1H), 6.65 (s, 1H), 4.61 (td,* J* = 8.2, 6.6 Hz, 1H), 3.78 (d,* J* = 5.9 Hz, 6H), 3.66 (s, 3H), 3.38–3.24 (m, 2H), 2.21 (s, 3H), 1.07 (s, 9H). ^13^C NMR (100 MHz, DMSO-d_6_): *δ*_C_ 177.5, 171.2, 169.0, 159.2, 151.2, 150.7, 129.2, 115.3, 114.7, 109.6, 103.6, 56.0, 55.5, 52.1, 50.4, 37.8, 27.5, 27.0, 16.2. MS (EI):* m*/*z* 404 (M + 1, 100). HRMS (ESI): Calcd for C_21_H_29_N_2_O_6_ [M + H]^+^: 405.2026; Found: 405.1703.

### 2.27. Experimental Procedure for the Preparation of Methyl 2-((tert-Butoxycarbonyl)amino)-3-(3-(3,6-dioxocyclohexa-1,4-dien-1-yl)isoxazol-5-yl)propanoate (**20a**)

To a solution of compound** 19a **(0.250 g, 0.61 mmol) in acetonitrile (10 mL) and H_2_O (2 mL), CAN (1.01 g, 1.84 mmol) was added and the reaction mixture was stirred at RT for 1 h. The progress of the reaction was monitored by TLC analysis (20% ethyl acetate/Pet ether). Then, water (10 mL) was added and extracted with ethyl acetate. The combined organic layer was washed with water, brined, and dried over anhydrous Na_2_SO_4_. Evaporation of the solvent in high vacuum gave the compound** 20a **(0.150 g, 64% yield) as yellow solid.

m.p. 110–112°C. IR (KBr, cm^−1^): 3359, 2979, 2955, 1749, 1688, 1524, 1283, 1163. ^1^H NMR (300 MHz, CDCl_3_): *δ* = 7.44–7.36 (m, 1H), 6.87 (d,* J* = 1.5 Hz, 2H), 6.69 (s, 1H), 5.22 (s, 1H), 4.68 (s, 1H), 3.80 (s, 3H), 3.53–3.25 (m, 2H), 1.44 (s, 9H). ^13^C NMR (100 MHz, CDCl_3_): *δ*_C_ 186.7, 185.0, 170.9, 169.3, 156.1, 154.9, 136.7, 136.5, 134.4, 133.3, 104.4, 80.4, 52.8, 52.0, 29.8, 28.2. MS (EI):* m*/*z* 376 (M + 1, 100). HRMS (ESI): Calcd for C_18_H_20_N_2_O_7_ [M + H]^+^: 377.1349; Found: 377.0531.

### 2.28. Experimental Procedure for the Preparation of Methyl 3-(3-(3,6-Dioxocyclohexa-1,4-dien-1-yl)isoxazol-5-yl)-2-pivalamidopropanoate (**20b**)

To a solution of compound** 19b **(0.2 g, 0.51 mmol) in acetonitrile (10 mL) and H_2_O (2 mL), CAN (0.560 g, 1.025 mmol) was added and the reaction mixture was stirred at RT for 1 h. The progress of the reaction was monitored by TLC analysis (30% ethyl acetate/pet ether). Then, water (10 mL) was added and extracted with ethyl acetate. The combined organic layer was washed with water, brined, and dried over anhydrous Na_2_SO_4_. Evaporation of the solvent in high vacuum gave the compound** 20b **(0.160 g, 86% yield) as yellow solid.

m.p. 107–109°C. IR (KBr, cm^−1^): 3312, 2961, 2872, 1752, 1662, 1639, 1534, 1287, 1206, 1093. ^1^H NMR (400 MHz, CDCl_3_): *δ* = 7.39 (s, 1H), 6.87 (s, 2H), 6.65 (s, 1H), 6.36 (d,* J* = 7.1 Hz, 1H), 4.90 (q,* J* = 5.7 Hz, 1H), 3.82 (s, 3H), 3.53 (dd,* J* = 15.4, 5.4 Hz, 1H), 3.37 (dd,* J* = 15.3, 5.3 Hz, 1H), 1.22 (s, 9H). ^13^C NMR (100 MHz, CDCl_3_): *δ*_C_ 186.7, 184.9, 178.2, 170.9, 169.2, 156.0, 136.7, 136.5, 134.3, 133.3, 104.5, 52.9, 50.8, 38.7, 29.0, 27.3. MS (EI):* m*/*z* 360 (M + 1, 100). HRMS (ESI): Calcd for C_18_H_21_N_2_O_6_ [M + H]^+^: 361.1400; Found: 361.1388.

### 2.29. Experimental Procedure for the Preparation of Methyl 2-((tert-Butoxycarbonyl)amino)-3-(3-(4-methyl-3,6-dioxocyclohexa-1,4-dien-1-yl)isoxazol-5-yl)propanoate (**20c**)

To a solution of compound** 19c** (0.2 g, 0.47 mmol) in acetonitrile (10 mL) and H_2_O (2 mL), CAN (0.52 g, 0.95 mmol) was added and the reaction mixture was stirred at RT for 1 h. The progress of the reaction was monitored by TLC analysis (30% ethyl acetate/pet ether). Then, water (10 mL) was added and extracted with ethyl acetate. The combined organic layer was washed with water, brined, and dried over anhydrous Na_2_SO_4_. Evaporation of the solvent in high vacuum gave the compound** 20c **(0.185 g, 86% yield) as yellow solid.

m.p. 89–93°C. IR (KBr, cm^−1^): 3379, 2977, 1742, 1695, 1654, 1524, 1239, 1170, 1040. ^1^H NMR (300 MHz, CDCl_3_): *δ* = 7.36 (s, 1H), 6.74–6.62 (m, 2H), 5.23 (d,* J* = 6.3 Hz, 1H), 4.68 (s, 1H), 3.80 (s, 3H), 3.39 (m, 2H), 2.25–1.99 (m, 3H), 1.44 (s, 9H). ^13^C NMR (100 MHz, CDCl_3_): *δ*_C_ 187.3, 185.2, 170.9, 169.1, 156.1, 154.9, 146.2, 134.2, 133.5, 133.4, 109.9, 104.5, 52.8, 52.0, 29.8, 28.2, 15.5. MS (EI):* m*/*z* 390 (M + 1, 100). HRMS (ESI): Calcd for C_19_H_23_N_2_O_7_ [M + H]^+^: 391.1505; Found: 391.1601.

### 2.30. Experimental Procedure for the Preparation of Methyl 3-(3-(4-Methyl-3,6-dioxocyclohexa-1,4-dien-1-yl)isoxazol-5-yl)-2-pivalamidopropanoate (**20d**)

To a solution of compound** 19d **(0.2 g, 0.49 mmol) in acetonitrile (10 mL) and H_2_O (2 mL), CAN (0.54 g, 0.99 mmol) was added and the reaction mixture was stirred at RT for 1 h. The progress of the reaction was monitored by TLC analysis (30% ethyl acetate/pet ether). After completion of the reaction, water (10 mL) was added and extracted with ethyl acetate thrice. The organic layers were combined, washed with water, brined, and dried over anhydrous Na_2_SO_4_. Evaporation of the solvent in high vacuum gave the compound** 20d **(0.175 g, 94% yield) as yellow solid.

m.p.118–120°C. IR (KBr, cm^−1^): 3351, 2958, 2872, 1735, 1657, 1524, 1230, 1042. ^1^H NMR (300 MHz, CDCl_3_): *δ* =7.36 (s, 1H), 6.70 (q,* J *= 1.6 Hz, 1H), 6.68 (s, 1H), 6.35 (d,* J *= 7.3 Hz, 1H), 4.89 (dt,* J *= 7.2, 5.2 Hz, 1H), 3.82 (s, 3H), 3.59–3.44 (m, 1H), 3.36 (dd,* J *= 15.2, 5.2 Hz, 1H), 2.11 (d,* J *= 1.7 Hz, 3H), 1.21 (s, 9H). ^13^C NMR (100 MHz, CDCl_3_): *δ*_C_ 187.3, 185.2, 178.2, 171.0, 169.0, 156.1, 146.2, 134.1, 133.5, 133.4, 104.6, 52.9, 50.8, 38.7, 29.0, 27.3, 15.5. MS (EI):* m*/*z* 374 (M + 1, 100). HRMS (ESI): Calcd for C_19_H_23_N_2_O_6_ [M + H]^+^: 375.1556; Found: 375.1468.

## 3. Results and Discussion

**Scheme 1:**
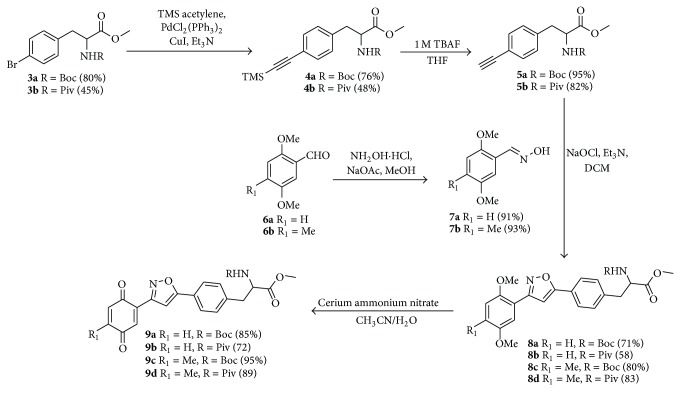
Synthesis of isoxazole tethered quinone phenylalanine hybrids.

Our proposed strategy was based on a simple and lucid cycloaddition reaction [42–45] of alkyne** 5** with oxime** 7** to prepare isoxazole-amino acid hybrids** 8 **(Scheme 1). The alkyne** 5** was prepared in five steps using the protocol developed by O'Donnell et al. starting from N-(diphenylmethylene) glycine methyl ester [46, 47] using appropriate benzyl or propargyl halides. Subsequent oxidation of the suitable placed methoxy group in the aromatic ring of compound** 8 **will provide isoxazole tethered quinone-amino acid hybrids** 9**.

To start with, the compound** 3a** or** 3b** was prepared according to literature procedure [48] starting from 4-bromobenzyl bromide. Then the compound** 3a** or** 3b** reacted with TMS-acetylene in the presence of CuI/Et_3_N/PdCl_2_(PPh_3_)_2_ in reflux condition to give compound** 4a** or** 4b** (Scheme 1). Then TMS group was deprotected using TBAF to give the key acetylenic amino acid ready for cycloaddition reaction. 2,5-Dimethoxy benzaldehyde** 6a** or** 6b** reacted with hydroxylamine hydrochloride (NH_2_OH*·*HCl) to produce the oxime derivative** 7a** or** 7b**. The compound** 7a** or** 7b** was subjected to the key 1,3-dipolar cycloaddition reaction with actetylenic amino acid** 5a** or** 5b** in the presence of NaOCl/Et_3_N in DCM as solvent. The isoxazoles** 8a–d** were smoothly formed using this Huisgen's one pot protocol. We did not observe the formation of any other isomer and nitrile oxide dimerization product in this reaction. Any undesired by-products resulting from aromatic halogenations reaction were not observed in our case.

**Figure 3:**
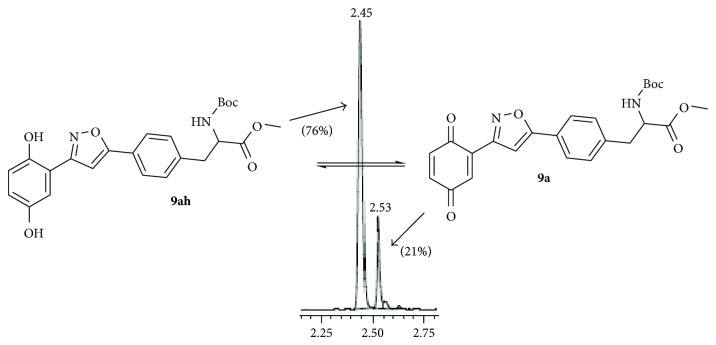
LC-MS analysis of compound** 9a** (showing the integrated percentage).

Then the compound** 8a **was oxidized with CAN to give the desired isoxazole tethered quinone-amino acid** 9a** in very good yield. The compound** 9a** was characterized by ^1^H-NMR, ^13^C-NMR, and HRMS. For example, the characteristic isoxazole proton at 7.1*δ* and quinone proton at 6.8*δ* in ^1^H-NMR confirms the oxidation of compound** 8a** to generate compound** 9a**. The two carbonyl peaks at 186.8 and 185.2*δ* in ^13^C-NMR spectrum validate the benzoquinone moiety. Using similar sequence (Scheme 1) the target compounds** 9b–d **(Table 1) were prepared and characterized by spectral data. The isoxazole tethered quinone-amino acids show equilibrium between hydroquinone (**9ah**) and benzoquinone (**9a**) in liquid chromatography mass spectrometry (LC-MS) analysis condition as shown in Figure 3. It may be possible that benzoquinone forms a reduced species during the ionization process in LC-MS condition [49].

**Scheme 2:**
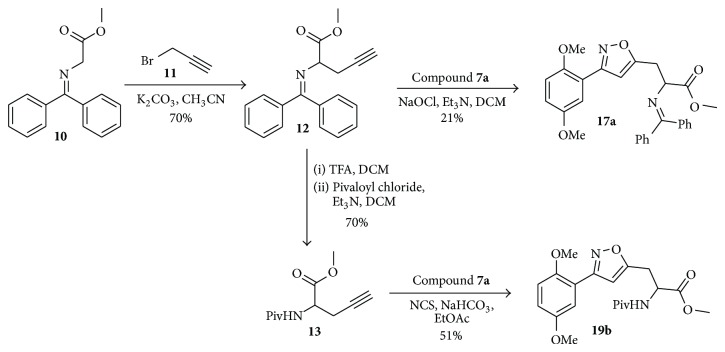
Synthesis of 3,5-disubstituted isoxazole derivative.

Encouraged by this result, we turn our attention to the preparation of propargyl amino acid as starting material. Various acetylene building blocks containing an amino acid moiety were prepared from Schiff-base N-(diphenylmethylene)glycine ester** 10** using the literature procedure [50]. Thus, alkylation of** 10** with propargyl bromide** 11** in the presence of K_2_CO_3_/CH_3_CN in reflux condition gave the propargylated derivative** 12**. Then the compound** 12** was subjected to 1,3-dipolar cycloaddition reaction with the oxime** 7a** in the presence of NCS/NaHCO_3_ in ethyl acetate as solvent to give very low yield of the compound** 17a** (Scheme 2). We tried couple of conditions to improve the yield of compound** 17a** but without any success. Maybe either steric factor or the instability of compound** 12** is the main reason behind the low yield of the cycloaddition product. Then we turn our attention to the compound** 13** which was prepared from compound** 12** by hydrolysis reaction followed by protection of the amino group. We tried several conditions (Table 2) to improve the yield of the compound** 19b** without any success.

**Scheme 3:**
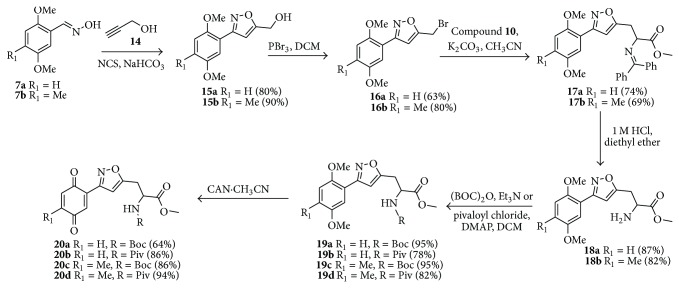
Synthesis of isoxazole tethered glycine quinone hybrids.

Alternatively we plan to introduce the amino acid at the end of reaction sequence to prepare the isoxazole tethered quinone-amino acid hybrid (Scheme 3). Thus, the oxime derivative** 7a** or** 7b** was subjected to cycloaddition reaction with propargyl alcohol** 14** using NCS/NaHCO_3_ condition in ethyl acetate as solvent. Under this reaction condition, the 3-aryl-isoxazole derivative** 15a** or** 15b** was prepared in good yield. Then the hydroxyl methyl group in the compound** 15a** or** 15b** was converted to bromo methyl group using PBr_3_/DCM condition to give the compound** 16a** or** 16b**.

Gratifyingly, the key step alkylation reaction of** 10** with compound** 16a** in the presence of K_2_CO_3_/CH_3_CN in reflux condition gave the 3,5-substituted isoxazole derivative** 17a **or** 17b**. The compound** 17a **was hydrolyzed in the presence of 1 N HCl/diethyl ether and the resulting amino ester** 18a** was protected with either Boc_2_O or pivaloyl chloride to obtain Boc derivative** 19a **or pivaloyl derivative** 19b**,  respectively. Then the compound** 19a** was oxidized with CAN to give the desired target isoxazole tethered quinone amino acid** 20a **in very good yield. The compound** 20a** was characterized by ^1^H-NMR, ^13^C-NMR, and HRMS spectral data. In a similar sequence the target compounds** 20b–d **were prepared and characterized by spectral data. The purity of the final isoxazole tethered quinone amino acids (Table 3) was obtained by LC-MS analysis which showed equilibrium between quinone and hydroquinone as observed earlier (Figure 3).

It is noteworthy to mention here that previously inaccessible quinone amino acids (**19a–d** and** 20a–d**) containing isoxazole moiety were synthesized in very good yield. As indicated in Table 3, the proting group (Boc or pivaloyl) has no effect on the yield of cycloaddition as well as oxidation reaction to give the isoxazole tethered quinone amino acids.

In conclusion, we have developed an efficient and simple method to synthesize isoxazole tethered quinone-amino acid hybrids using 1,3-dipolar cycloaddition and oxidation reactions as key steps in good yields. We believe that this methodology will find a widespread application for the synthesis of 2-aryl-benzoquinone and its derivatives. Further application of this methodology for the synthesis of isoxazole tethered quinone-peptide hybrid as well as preparation of tetrazole tethered quinone-amino acid hybrid is undergoing in our group.

## Figures and Tables

**Table 1:** The yields and purity of the novel isoxazole-phenyl alanine and isoxazole tethered quinone-phenyl alanine hybrids.

Isoxazole amino acid	Yield (%)	Quinone isoxazole amino acid	Yield (%)	Purity^a^
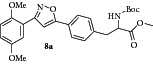	71	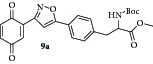	85	97
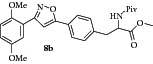	58	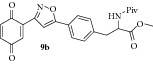	72	92
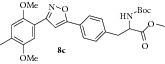	80	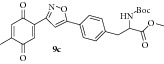	95	96
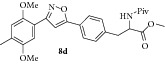	83	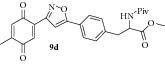	89	94

^a^Combined purity of hydroquinone and quinone by LC-MS analysis.

**Table 2:** The yields of the compound **19b** using various 1,3-cycloaddition reaction conditions.

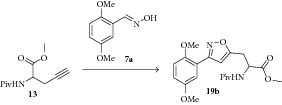			

Entry	Reaction conditions	Time	Yield (%)^a^

1	NCS, NaHCO_3_, EtOAc	16 h	43
2	(Bu_3_Sn)_2_O, t-BuOCl, DCM [36]	16 h	11
3	NaOCl, Et_3_N, DCM	16 h	26
4	NCS, K_2_CO_3_, EtOAc, H_2_O	16 h	49
5	CuSO_4_·5H_2_O, L-Ascorbate, NaHCO_3_, t-BuOH, H_2_O [37]	12 h	30
6	NaI, 2,6-Lutidine, t-BuOCl, dioxane [38]	12 h	30
7	HTIB, CH_3_CN [39]	12 h	0
8	NBS, Et_3_N, DMF [40]	18 h	11
9	CrO_2_, CH_3_CN [41]	12 h	18

^a^Determined by analysis of the crude reaction mixture by analytical LC/MS.

HTIB: hydroxy(tosyloxy)iodobenzene; NCS: N-chloro succinamide; NBS: N-bromosuccinamide.

**Table 3:** The yields and purity of the novel isoxazole-glycine and isoxazole tethered quinone-glycine hybrids.

Isoxazole amino acid	Yield (%)	Quinone isoxazole amino acid	Yield (%)	Purity^a^
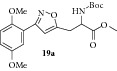	95	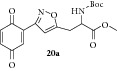	65	90
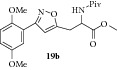	78	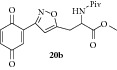	86	91
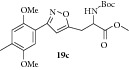	95	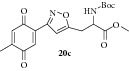	86	90
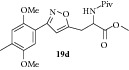	82	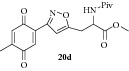	94	94

^a^Combined purity of hydroquinone and quinone by LC-MS analysis.
